# Health-related quality of life with bemarituzumab plus mFOLFOX6 in patients with FGFR2b-overexpressing, advanced gastric or gastroesophageal junction cancer

**DOI:** 10.1016/j.esmogo.2024.100095

**Published:** 2024-10-08

**Authors:** Z.A. Wainberg, P.C. Enzinger, S. Qin, K. Yamaguchi, J. Wang, X. Zhou, A. Gnanasakthy, K. Taylor, A. Yusuf, I. Majer, A. Jamotte, Y.-K. Kang

**Affiliations:** 1 Department of Medicine, University of California Los Angeles Medical Center, Los Angeles; 2 Department of Medicine, Dana Farber Cancer Institute, Boston, USA; 3 Nanjing Tianyinshan Hospital, the 1st Affiliated Hospital of China Pharmaceutical University, Nanjing, China; 4 Gastroenterological Chemotherapy Department, The Cancer Institute Hospital of JFCR, Koto-Ku, Tokyo, Japan; 5 RTI Health Solutions, Research Triangle Park, USA; 6 Global Biostatistics, Amgen Ltd., London, UK; 7 Global Medical Affairs, Amgen Inc., Thousand Oaks, USA; 8 Global HEOR, Amgen (Europe) GmbH, Rotkreuz, Switzerland; 9 Asan Medical Centre, University of Ulsan College of Medicine, Seoul, South Korea

**Keywords:** bemarituzumab, FGFR2b, gastric cancer, health-related quality of life, mFOLFOX6, targeted therapy

## Abstract

**Background:**

In the phase II, randomized, double-blind FIGHT trial (NCT03694522), treatment with bemarituzumab plus mFOLFOX6 resulted in improvements in progression-free survival and overall survival relative to mFOLFOX6 alone in previously untreated locally advanced or metastatic gastric or gastroesophageal junction cancer with fibroblast growth factor receptor 2b overexpression. Using data from the final analysis, we analyzed patient-reported outcomes (PROs) to evaluate the impact of adding bemarituzumab to mFOLFOX6 on health-related quality of life (HRQoL).

**Materials and methods:**

Patients were randomized 1 : 1 to bemarituzumab plus mFOLFOX6 (*n* = 77) or placebo plus mFOLFOX (*n* = 78). European Organisation for Research and Treatment of Cancer Core 30-item Quality of Life (EORTC QLQ-C30) and the EuroQol EQ-5D-5L questionnaires were administered at baseline, week 6, every 8 weeks thereafter, and at end-of-treatment visit. Least-squares mean changes from baseline in PRO scale scores were estimated using mixed models for repeated measures; time to deterioration and improvement were assessed using Cox proportional hazards models. Analyses were exploratory *post hoc*.

**Results:**

PRO scale scores at baseline and compliance rates across PRO assessments over time were similar between the bemarituzumab and placebo arms. Least-squares mean changes from baseline on key EORTC QLQ-C30 scales (global health status/QoL, physical functioning, fatigue, nausea and vomiting, and appetite loss) and the EQ-5D-5L visual analog scale were similar over time between treatment arms. Analyses of time to deterioration, sustained deterioration, and improvement suggested similar HRQoL between treatment arms.

**Conclusions:**

Treatment with bemarituzumab plus mFOLFOX6 was associated with sustained HRQoL relative to mFOLFOX6 alone.

## Highlights

• We assessed the impact of adding bemarituzumab to mFOLFOX6 on QoL.
• Change from baseline and time to deterioration and improvement were evaluated.
• Bemarituzumab/mFOLFOX6 was associated with sustained QoL relative to mFOLFOX6 alone.

## Introduction

Worldwide, gastric cancer, including gastroesophageal junction cancer (G/GEJC), is the fifth most commonly diagnosed cancer and the fourth leading cause of cancer-related death.^1^^,^^2^ G/GEJC is typically diagnosed at an advanced stage when prognosis is poor and patients experience significant impacts in aspects of health-related quality of life (HRQoL) such as fatigue, pain, loss of appetite, or impairments in physical functioning.^3^^,^^4^ Survival in patients with HER2-negative, locally advanced unresectable or metastatic G/GEJC who receive standard first-line platinum-fluoropyrimidine chemotherapy is about 1 year.^5^, ^6^, ^7^ The addition of an immune checkpoint inhibitor, such as nivolumab, pembrolizumab, and tislelizumab, to standard chemotherapy can improve survival.^8^, ^9^, ^10^, ^11^ Recent phase III studies also demonstrated that in patients with CLDN18.2-positive cancer, the biomarker-targeted agent zolbetuximab may extend survival when combined with standard chemotherapy.^12^^,^^13^ Despite these improvements, patients with advanced G/GEJC continue to experience considerable unmet medical needs; therefore, novel and effective therapies are urgently needed.

Approximately 20%-30% of patients with advanced HER2-non-positive G/GEJC overexpress the fibroblast growth factor receptor 2b (FGFR2b) protein, depending on the cut-off point for immunohistochemistry (IHC) staining.^14^ Although the prognostic relevance of FGFR2b overexpression has not yet been fully established, published evidence suggests that it may be associated with poor prognosis.^15^ Bemarituzumab is a first-in-class monoclonal antibody that has shown inhibition of FGFR2b signaling and enhanced antibody-dependent cell-mediated cytotoxicity against tumor cells that express FGFR2b.^16^ The phase II, randomized, double-blind FIGHT (NCT03694522) study evaluated the efficacy and safety of bemarituzumab plus modified FOLFOX6 (infusional 5-fluorouracil, leucovorin, and oxaliplatin; mFOLFOX6) chemotherapy versus placebo plus mFOLFOX6 in previously untreated patients with HER2-non-positive advanced G/GEJC. Eligible patients had positive FGFR2b status, i.e. exhibited any moderate (2+) to strong (3+) membranous staining in any tumor cells based on centrally assessed IHC and/or tested positive for *FGFR2* amplification based on plasma next-generation sequencing of cell-free circulating tumor DNA.^14^ At the final analysis after a minimum follow-up of 24 months (data cut-off 13 May 2022), bemarituzumab plus mFOLFOX6 showed improved progression-free survival (PFS) (median PFS 9.5 versus 7.4 months, hazard ratio [HR] 0.72; 95% confidence interval [CI] 0.49-1.08) and overall survival (OS) (median OS 19.2 versus 13.5 months, HR 0.77; 95% CI 0.52-1.14).^17^ The treatment benefit was more pronounced in the subgroup of patients with 2+/3+ FGFR2b IHC staining intensity in ≥10% of tumor cells, for whom median PFS with bemarituzumab plus mFOLFOX6 was 14.0 months versus 7.3 months with placebo plus mFOLFOX6 (HR 0.43; 95% CI 0.26-0.73) and median OS was 24.7 months versus 11.1 months, respectively (HR 0.52; 95% CI 0.31-0.85). In FIGHT, grade ≥3 treatment-emergent adverse events occurred in 83% and 75% of patients in the bemarituzumab plus mFOLFOX6 and placebo plus mFOLFOX6 groups, respectively. All-grade corneal events (adverse events of special interest) occurred in 67% of patients in the bemarituzumab group and 10% in the placebo group; grade ≥3 corneal events were reported in 28% of patients in the bemarituzumab group and in no patients in the placebo group.

In addition to evaluating the survival and safety outcomes associated with bemarituzumab, it is important to understand the impact of treatment on patients’ symptom experiences and HRQoL. The objective of this analysis was to assess the impact of bemarituzumab plus mFOLFOX6 versus placebo plus mFOLFOX6 on HRQoL in FGFR2b-positive patients with previously untreated advanced or metastatic G/GEJC, based on the patient-reported outcome (PRO) measures included in the FIGHT study.

## Materials and methods

### Study design

The trial design, protocol, and primary results of the FIGHT study have been reported previously.^14^^,^^17^ Briefly, eligible participants were patients aged 18 years and older with HER2 non-positive, FGFR2b-positive, histologically confirmed, unresectable, locally advanced or metastatic G/GEJC not amenable to curative therapy. Eligible patients were stratified by geographic region, prior treatment status, and administration of a single dose of mFOLFOX6 before enrollment. Patients were recruited from 164 clinical sites across 18 countries and were randomized 1 : 1 to receive 15 mg/kg of body weight bemarituzumab every 2 weeks (with a single additional bemarituzumab 7.5 mg/kg dose on cycle 1 day 8) or matched placebo intravenously. All patients also received mFOLFOX6 (oxaliplatin 85 mg/m^2^, leucovorin 400 mg/m^2^, and 5-fluorouracil as a 400 mg/m^2^ bolus followed by 2400 mg/m^2^ over ∼48 h) intravenously every 2 weeks. Patients were treated until disease progression (defined by RECIST version 1.1), unacceptable toxicity, withdrawal of consent, or death. The primary endpoint was PFS; secondary endpoints included OS, objective response rate, and incidence of adverse events. PROs were assessed as exploratory endpoints.

All procedures followed were in accordance with the ethical standards of the responsible committee on human experimentation (institutional and national) and with the Helsinki Declaration of 1964 and later versions. Informed consent to be included in the study, or the equivalent, was obtained from all patients.

### Patient-reported outcome measures

Two PRO measures were administered in FIGHT: the European Organisation for Research and Treatment of Cancer Core 30-item Quality of Life Questionnaire (EORTC QLQ-C30) and the EuroQol EQ-5D-5L questionnaire (Supplementary Appendix, available at https://doi.org/10.1016/j.esmogo.2024.100095).^18^^,^^19^ PRO questionnaires were administered at screening, before receiving study drug at cycle 4 (week 6), every 8 weeks thereafter until treatment discontinuation, and at the end-of-treatment visit (i.e. last treatment administration).

### Statistical analyses

The analyses used data from the most mature, final analysis of the FIGHT study (data cut-off 13 May 2022). Compliance rates at each study visit were defined as the percentage of subjects completing PRO assessments relative to all subjects who were expected to have a visit. PRO assessment at baseline was defined as the assessment before the first treatment administration. To account for delayed and/or missed treatment administration and varying end-of-treatment time points, PRO visit dates were classified into the analytical windows labeled by the week of the target assessment date. An overview of the analytical windows for the PROs is provided in Supplementary Table S1, available at https://doi.org/10.1016/j.esmogo.2024.100095.

For each scale of the EORTC QLQ-C30 questionnaire and the visual analog scale (VAS) of the EQ-5D-5L questionnaire, least-squares (LS) mean change from baseline in PRO scores for the treatment arms and the difference between the treatment arms were estimated using linear mixed models for repeated measures. The models included treatment indicator, baseline score, analysis visit (as a categorical variable), treatment-by-analysis visit interaction, and randomization stratification factors, i.e. geographic region (US/EU or China or rest of Asia), prior treatment status (*de novo* or adjuvant/neoadjuvant), and administration of a single dose of mFOLFOX6 before enrollment (yes or no) as fixed-effect covariates. Heterogeneous Toeplitz covariance structure was used in the model to account for the within-patient correlations. Analyses were conducted on patients with baseline and at least one post-baseline PRO score, and included study visits with at least 10 patients with evaluable data in each treatment arm. The model assumed missing data were missing at random.

Time to deterioration and time to improvement in PRO scale scores were also estimated. Time to deterioration and time to improvement were defined as the time from baseline until first PRO assessment with a decrease or increase, depending on scale directions, in score that reached at least the clinically meaningful change threshold, or death (for deterioration) if occurring within 12 weeks of the last PRO visit. Patients who did not experience a deterioration (or improvement), or, for deterioration, died beyond 12 weeks of the last PRO visit were censored at the date of the last PRO questionnaire completion (i.e. date of the last non-missing value). The clinically meaningful change threshold was defined as a within-subject change of 10 points for the EORTC QLQ-C30 and 7 points for the EQ-5D VAS.^20^^,^^21^ Time to sustained deterioration also was estimated and was defined similarly to time to deterioration but requiring (i) no subsequent recovery above the clinically meaningful change threshold and (ii) confirmation with a subsequent PRO assessment with deterioration or death within 12 weeks of first deterioration. To estimate the difference in the risk of deterioration and improvement across the treatment arms, time-to-event analyses were conducted using Cox proportional hazards models including randomization stratification factors and treatment as covariates. The median time to (sustained) deterioration and improvement was estimated using the Kaplan–Meier method. Subjects who had no baseline or no post-baseline assessments or could not experience deterioration or improvement on a given PRO scale score because that score was too high or too low at baseline were censored with a time-to-event duration of 1 day for that scale.

Subgroup analyses were carried out in patients with an IHC 2+/3+ FGFR2b overexpression in ≥10% of tumor cells, as well as in patients with Asian and non-Asian geographical locations. Analyses were conducted to assess differences in change from baseline between the treatment arms in PRO scales that were considered most important and directly relevant to patients with advanced gastric cancer (EORTC QLQ-C30 global health status/QoL, physical functioning, fatigue, nausea and vomiting, appetite loss, and EQ-5D-5L VAS). Time-to-deterioration and time-to-improvement analyses were not conducted because it was hypothesized that due to the smaller sample sizes and generally large patient-level variability in these outcomes, results would be associated with large uncertainty and difficult to interpret.

All analyses were considered as exploratory and no adjustments for multiple testing or estimation were used. An estimate of the difference in change from baseline between the treatment arms with a 95% CI not including 0 was considered as different. Similarly, an HR estimated to compare time to deterioration or time to improvement across the treatment arms with a 95% CI not including 1 was considered as different. The analyses were conducted using SAS version 9.4 (SAS Institute, Cary, NC).

## Results

### Baseline characteristics and PRO compliance

The study randomized 155 patients: 78 in the group treated with bemarituzumab and mFOLFOX6 (bemarituzumab arm) and 77 in the group treated with placebo plus mFOLFOX6 (placebo arm). Demographic characteristics and descriptive PRO scale scores at baseline were well balanced between treatment arms (Table 1). The median duration of treatment was 24.0 weeks (range 2.0-96.9 weeks) in the bemarituzumab arm and 26.0 weeks (range 2.0-130.7 weeks) in the placebo arm. Almost all patients had discontinued treatment by the data cut-off date.

**Table 1:** Patient characteristics and descriptive patient-reported outcome scale scores at baseline

Characteristic	Bemarituzumab + mFOLFOX6 (*N* = 77)	Placebo + mFOLFOX6 (*N* = 78)
Patient characteristics		
Age, mean (SD)	58.0 (11.1)	59.1 (12.0)
Male, *n* (%)	52 (67.5)	59 (75.6)
Race, *n* (%)		
Asian	45 (58.4)	44 (56.4)
American Indian or Alaska native	0	1 (1.3)
Black	0	1 (1.3)
Other^a^	2 (2.6)	1 (1.3)
White	30 (39.0)	31 (39.7)
ECOG performance status		
0	25 (32.5)	28 (35.9)
1	52 (67.5)	50 (64.1)
Site of primary cancer, *n* (%)		
Gastric adenocarcinoma	66 (85.7)	71 (91.0)
Gastroesophageal junction adenocarcinoma	11 (14.3)	7 (9.0)
Metastatic disease, *n* (%)	73 (94.8)	66 (84.6)
Tumor histology, *n* (%)		
Diffuse	28 (36.4)	26 (33.3)
Intestinal	16 (20.8)	15 (19.2)
Mixed	5 (6.5)	12 (15.4)
Unknown	28 (36.4)	25 (32.1)
mFOLFOX6 therapy before randomization, *n* (%)	35 (45.5)	36 (46.2)
Prior neoadjuvant or adjuvant therapy, *n* (%)	14 (18.2)	13 (16.7)
FGFR2b expression, *n* (%)		
IHC staining score of 2+ or 3+ in ≥10% of tumor cells	46 (59.7)	52 (66.7)
**PRO scale scores at baseline**	**(*n* = 75-76)**	**(*n* = 72-73)**
EORTC QLQ-C30 scale scores, mean (SD)^b^		
Global health status/QoL	63.9 (20.4)	59.1 (21.5)
Physical functioning	81.3 (17.7)	77.2 (20.3)
Role functioning	78.5 (25.4)	78.3 (25.0)
Emotional functioning	77.0 (20.0)	77.4 (21.7)
Cognitive functioning	89.5 (14.6)	88.9 (17.7)
Social functioning	74.6 (25.9)	78.0 (21.8)
Fatigue	31.1 (20.9)	36.5 (24.8)
Nausea and vomiting	15.6 (19.5)	17.1 (24.7)
Pain	22.6 (21.0)	26.3 (25.0)
Dyspnea	9.2 (18.5)	13.2 (24.0)
Insomnia	22.4 (29.0)	29.2 (33.8)
Appetite loss	28.9 (28.5)	31.5 (31.4)
Constipation	19.6 (26.3)	20.1 (30.8)
Diarrhea	11.0 (20.6)	11.6 (21.8)
Financial difficulties	21.1 (25.4)	23.6 (30.9)
EQ-5D-5L scores, mean (SD)^c^		
EQ-5D VAS	70.5 (20.1)	68.9 (19.6)

ctDNA, circulating tumor DNA; ECOG, Eastern Cooperative Oncology Group; EORTC QLQ-C30, European Organisation for Research and Treatment of Cancer Quality of Life Questionnaire; FGFR2b, IIIb splice isoform of the fibroblast growth factor receptor 2; IHC, immunohistochemistry; mFOLFOX6, modified FOLFOX (infusional 5-FU, leucovorin, and oxaliplatin); PRO, patient-reported outcome; QoL, quality of life; SD, standard deviation; VAS, visual analog scale.

a Race and/or ethnicity not further specified.

b EORTC QLQ-C30 scale scores range from 0 to 100, with higher scores indicating better HRQoL on the global and functional scales and greater symptom burden on the symptom scales.

c EQ-5D VAS score ranges from 100, ‘the best health you can imagine’, to 0, ‘the worst health you can imagine’.

A total of 76 patients (99%) in the bemarituzumab arm and 73 patients (94%) in the placebo arm completed the EORTC QLQ-C30 and the EQ-5D-5L at baseline. Compliance rates for the EORTC QLQ-C30 PRO measure were similar between treatment arms and remained high through week 54 (71%-95% in the bemarituzumab arm and 73%-92% in the placebo arm) but were variable in later cycles when the number of patients was small (<15% of the intention-to-treat population treated) (Table 2). Compliance rates for the EQ-5D-5L questionnaire were similar to those for the EORTC QLQ-C30 (Supplementary Table S2, available at https://doi.org/10.1016/j.esmogo.2024.100095).

**Table 2:** Compliance for the EORTC QLQ-C30 questionnaire

Visit	Total (*N* = 155)	Bemarituzumab arm (*N* = 77)	Placebo arm (*N* = 78)
**EORTC QLQ-C30**			
Baseline	149 (96%)	76 (99%)	73 (94%)
Week 6	127/152 (84%)	65/75 (87%)	62/77 (81%)
Week 14	105/119 (88%)	54/61 (89%)	51/58 (88%)
Week 22	73/101 (72%)	35/49 (71%)	38/52 (73%)
Week 30	58/74 (78%)	34/37 (92%)	24/37 (65%)
Week 38	36/49 (73%)	19/26 (73%)	17/23 (74%)
Week 46	30/38 (79%)	18/22 (82%)	12/16 (75%)
Week 54	29/31 (94%)	18/19 (95%)	11/12 (92%)
Week 62	17/23 (74%)	10/13 (77%)	7/10 (70%)
Week 70	13/18 (72%)	8/10 (80%)	5/8 (63%)
Week 78	10/15 (67%)	4/8 (50%)	6/7 (86%)
Week 86	11/13 (85%)	5/6 (83%)	6/7 (86%)
Week 94	7/12 (58%)	6/6 (100%)	1/6 (17%)
Week 102	7/8 (88%)	4/4 (100%)	3/4 (75%)
Week 110	5/5 (100%)	4/4 (100%)	1/1 (100%)
Week 118	4/5 (80%)	3/4 (75%)	1/1 (100%)
Week 126	4/5 (80%)	4/4 (100%)	0/1 (0%)
Week 134	3/3 (100%)	3/3 (100%)	0
Week 142	1/2 (50%)	1/2 (50%)	0
Week 150	1/1 (100%)	1/1 (100%)	0
End of treatment	62/152 (41%)	28/75 (37%)	34/77 (44%)

Note: Less than 15% of the ITT population was treated hence expected to have a PRO assessment after week 54. Compliance rate (at week X) = (number of patients who complete at least one item in the subscale of the questionnaire at week X/*N*) ∗ 100%. *N* = number of eligible patients who are expected to complete the PRO assessment per protocol at week X, i.e. still on study and still alive, excluding the patients who are missing by design, such as death, disease progression, study discontinuation, etc.

EORTC QLQ-C30, European Organisation for Research and Treatment of Cancer Quality of Life Questionnaire; ITT, intention-to-treat; PRO, patient-reported outcome.

### Change from baseline in PRO scores during treatment

**Figure 1:**
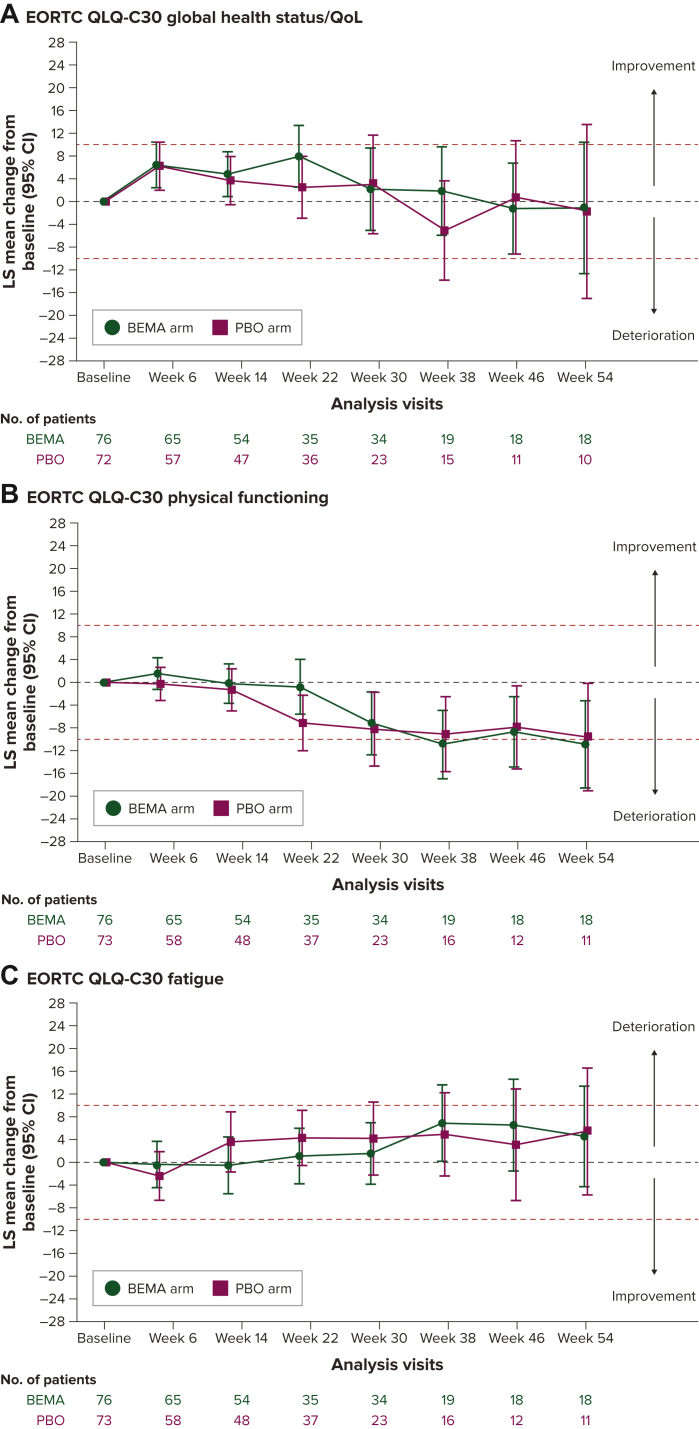

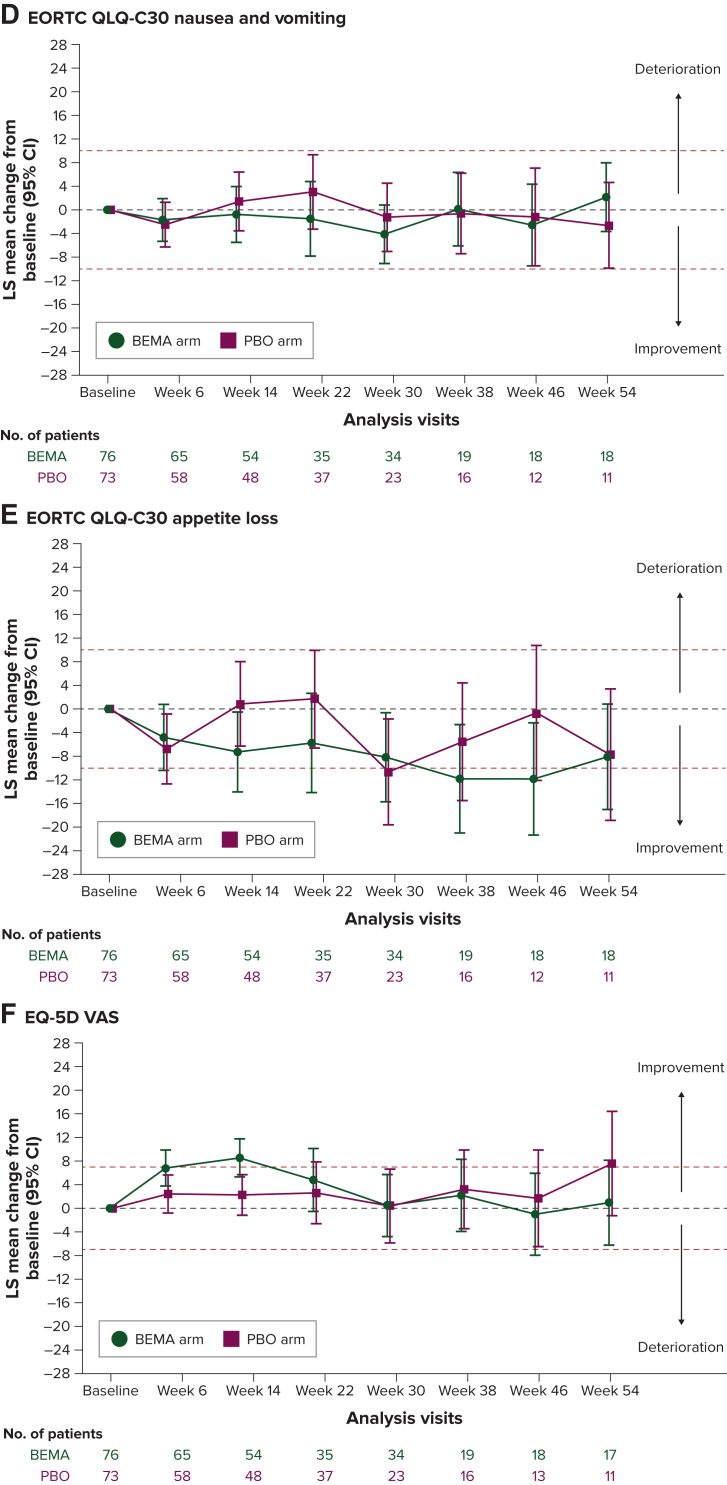
**Least-squares mean changes from baseline in PRO scores.** (A) EORTC QLQ-C30 global health status/QoL. (B) EORTC QLQ-C30 physical functioning. (C) EORTC QLQ-C30 fatigue. (D) EORTC QLQ-C30 nausea and vomiting. (E) EORTC QLQ-C30 appetite loss. (F) EQ-5D VAS. BEMA, bemarituzumab; CI, confidence interval; EORTC QLQ-C30, European Organisation for Research and Treatment of Cancer Quality of Life Questionnaire; HRQoL, health-related quality of life; LS, least square; PBO, placebo; VAS, visual analog scale. Red dotted lines indicate thresholds for meaningful change from baseline. Visits after week 54 were excluded because of the small sample sizes (<10 patients in each treatment group). EORTC QLQ-C30 scale scores range from 0 to 100, with higher scores indicating better HRQoL on the global and functional scales and greater symptom burden on the symptom scales. EQ-5D VAS score ranges from 100, ‘the best health you can imagine’, to 0, ‘the worst health you can imagine’.

The results shown in Figure 1 focus on the EORTC QLQ-C30 scales considered most clinically relevant to patients with advanced gastric cancer. The estimated LS mean change scores for global health status/QoL as measured by the EORTC QLQ-C30 and respondents’ health status as measured by the EQ-5D-5L VAS suggested that HRQoL was generally maintained from baseline at most assessment time points through week 54 for both treatment arms (Figures 1A-F) and improved on the EQ-5D-5L VAS for the bemarituzumab arm at weeks 6 and 14, with the mean change from baseline scores different from 0 (the 95% CI did not overlap with 0) and reaching the clinically meaningful threshold. The estimated LS mean change scores for EORTC QLQ-C30 physical functioning worsened, approaching the clinically meaningful threshold, after week 14 for the placebo arm and after week 22 for the bemarituzumab arm. Fatigue and nausea as well as vomiting scores were generally stable over time for both arms. Appetite loss reached the clinically meaningful threshold for improvement for the bemarituzumab arm at weeks 38 and 46 and the placebo arm at week 30. The 95% CIs suggested no differences between treatment arms, except for the EQ-5D-5L VAS at week 14 favoring the bemarituzumab arm [LS mean difference 6.3, 95% CI 1.5-11.0; Supplementary Table S3, available at https://doi.org/10.1016/j.esmogo.2024.100095)].

LS mean changes from baseline for other EORTC QLQ-C30 scale scores are provided in Supplementary Table S3, available at https://doi.org/10.1016/j.esmogo.2024.100095. Based on the 95% CIs, patients in the bemarituzumab arm experienced improvements compared with patients in the placebo arm at some time points in role functioning (weeks 6 and 14), emotional functioning (weeks 6, 22, 30, and 38), and social functioning (weeks 6 and 14). There were no differences between treatment arms observed for the other symptom scales except for constipation at early visits (up to week 22) and diarrhea at week 46, where patients in the placebo arm, but not the bemarituzumab arm, had worsening in scores.

### Subgroup analyses

Estimated LS mean changes from baseline in EORTC QLQ-C30 global health status/QoL, physical functioning, fatigue, nausea and vomiting, appetite loss, and EQ-5D-5L VAS scores in the subgroups of patients with FGFR2b overexpression ≥10% and geographical location (Asia, non-Asia) were largely consistent with the overall population (see results with respect to the global health status/QoL in Supplementary Figure S1, available at https://doi.org/10.1016/j.esmogo.2024.100095). For bemarituzumab-treated patients (*n* = 46) in the FGFR2b ≥10% subgroup, improvements relative to the placebo arm (*n* = 52) were observed for physical functioning at week 22 [difference in LS mean change between treatment arms, 10.3 (95% CI 2.0-18.6)], fatigue at week 22 [−8.9 (95% CI −17.6 to −0.2)], appetite loss at week 14 [−14.0 (95% CI −26.5 to −1.6)], and EQ-5D-5L VAS at week 14 [6.8 (95% CI 0.6-12.9)] and week 22 [10.4 (95% CI 0.1-20.7)]. No differences were observed on any scores between treatment arms for the subgroups of patients in Asian or non-Asian geographical location, although results were associated with larger uncertainty due to the low number of patients in the non-Asian subgroup (*n* = 59 across arms at baseline and further reduced after treatment initiation).

### Time to deterioration and time to improvement

**Figure 2:**
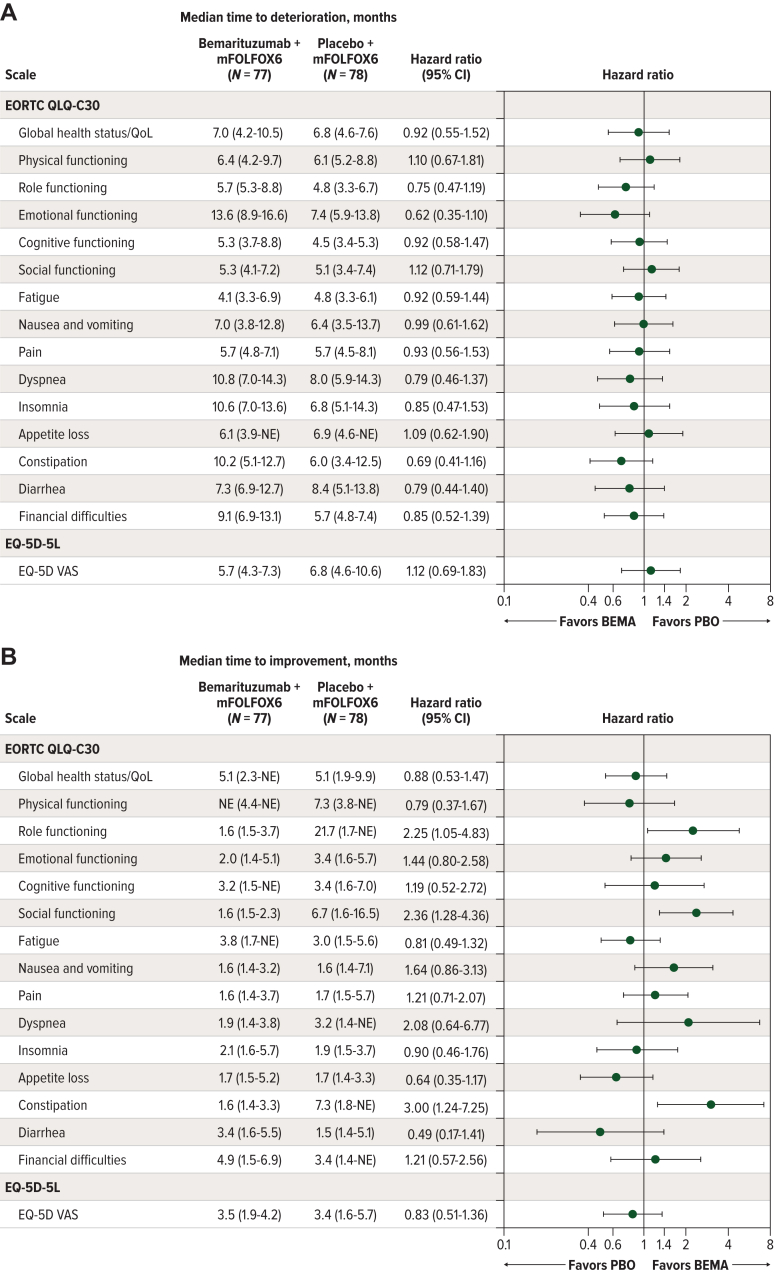
**Time to deterioration and time to improvement.** (A) Time to deterioration. (B) Time to improvement. Hazard ratios are presented in log scale plots. BEMA, bemarituzumab; CI, confidence interval; EORTC QLQ-C30, European Organisation for Research and Treatment of Cancer Quality of Life Questionnaire; PBO, placebo; QoL, quality of life; VAS, visual analog scale.

The proportion of patients meeting the criteria for first deterioration and sustained deterioration events were similar between the treatment arms for each scale and varied from 33.3% (insomnia) to 58.4% (fatigue) with respect to first deterioration and from 5.2% (insomnia) to 30.8% (role functioning) with respect to sustained deterioration. In general, only a small proportion of patients reported PROs with sustained deterioration; therefore, the median time to these events were not estimable for most scales. The median time to first deterioration varied by scale, ranging from 4.1 to 13.6 months in the bemarituzumab arm and from 4.5 to 8.4 months in the placebo arm (Figure 2A). For most scales, the estimated HRs showed a similar or smaller risk of deterioration for patients in the bemarituzumab arm than in the placebo arm, especially for role functioning, emotional functioning, and constipation. HRs ranged from 0.62 in the emotional functioning scale to 1.12 in the social functioning scale, and all had a 95% CI that included 1.00, suggesting no differences between the treatment groups (Figure 2A). Similarly, the estimated HRs for time to sustained deterioration suggested a similar or numerical trend favoring the bemarituzumab arm on most scales. The HRs ranged from 0.43 (appetite loss scale) to 1.40 (cognitive functioning scale) with a 95% CI that included 1.00 (Supplementary Figure S2, available at https://doi.org/10.1016/j.esmogo.2024.100095).

The proportion of patients experiencing improvement on the PRO scales varied from 12.8% (dyspnea) to 48.1% (EQ-5D-5L VAS), which was generally similar between the treatment arms but was notably higher in the bemarituzumab arm for the role functioning (32.9% versus 17.9%), social functioning (46.1% versus 29.5%), nausea and vomiting (39.5% versus 24.4%), and constipation (30.3% versus 12.8%) scales. The median time to improvement ranged across the different scales from 1.6 months (social functioning scale) to 5.1 months (global health status/QoL scale) in the bemarituzumab arm, except that it was not reached for physical functioning. In the placebo arm, the median time to improvement ranged from 1.5 months (diarrhea scale) to 21.7 months (role functioning scale) months. The 95% CIs of the HR indicated no differences between treatment arms for most scores, and there was an estimated benefit for bemarituzumab on the role functioning, social functioning, and constipation scales (Figure 2B).

## Discussion

In FIGHT, treatment of FGFR2b-positive advanced G/GEJC patients with bemarituzumab plus mFOLFOX6 resulted in clinically meaningful improvements in PFS and OS relative to placebo plus mFOLFOX6.^14^^,^^17^ The current PRO analyses suggest that patients in the bemarituzumab and placebo arms had similar PRO scores over time on the available scales that were considered most relevant in advanced G/GEJC (EORTC QLQ-C30 global health status/QoL, physical functioning, fatigue, nausea and vomiting and appetite loss, and EQ-5D-5L VAS), with no meaningful differences between treatment arms. Similarly, time to deterioration, time to sustained deterioration, and time to improvement results were generally similar between treatment arms, although the 95% CIs indicated a benefit for bemarituzumab with respect to time to improvement on the EORTC QLQ-C30 role functioning, social functioning, and constipation scales. Results of these analyses are consistent with previous findings from recent trials in the first-line treatment of advanced gastric cancer demonstrating that HRQoL may be maintained with the addition of an immunotherapy or targeted therapy to chemotherapy compared with chemotherapy alone.^22^, ^23^, ^24^, ^25^

In a prespecified exploratory analysis of FIGHT, the benefits in PFS (median months, 14.0 versus 7.3), OS (median months, 24.7 versus 11.1), and ORR (56.5% versus 36.5%) with bemarituzumab plus mFOLFOX6 versus placebo plus FOLFOX6 were more pronounced in patients with IHC 2+/3+ FGFR2b overexpression in ≥10% of tumor cells than in the overall patient population.^17^ Analyses of PROs in this subgroup of patients confirmed that the addition of bemarituzumab to chemotherapy did not increase treatment-related symptom burden or negatively affect HRQoL compared with chemotherapy alone, despite a longer duration of the combination therapy, while improving clinical outcomes. Further, the FIGHT safety analyses revealed a higher incidence of corneal adverse events and stomatitis in bemarituzumab-treated patients (corneal adverse events: 67.1% all-grade with a median onset of 16.9 weeks; stomatitis: 34.2% all-grade) than in placebo-treated patients (corneal adverse events: 10.4% with a median onset of 11.6 weeks; stomatitis: 9.2% all-grade).^14^ While there were no PRO measures included in FIGHT that could have assessed the patients’ overall side-effect burden and the impact of certain corneal adverse event symptoms or stomatitis on usual activities specifically, PROs that assessed the overall health status of patients implicitly capturing the impact of adverse events (e.g. GHS/QoL or EQ-5D-5L VAS, pain, appetite loss) were similar for both treatment groups. This may be explained by the study design stipulating the discontinuation of treatment for any grade 2 and grade 3 corneal treatment-emergent adverse event that was not resolved or improved to grade 1 within 28 days of adverse event onset, the effective management of corneal adverse event by adjusting the dose and administration schedule of treatments,^14^ or a patient’s tolerability to the adverse event.

The benefit of bemarituzumab on OS is currently being further assessed in patients with previously untreated locally advanced unresectable or metastatic HER2-negative G/GEJC and FGFR2b overexpression in the randomized, multicenter, double-blind phase III FORTITUDE-101 (NCT05052801) and FORTITUDE-102 (NCT05111626) trials.^26^^,^^27^ FORTITUDE-101 will evaluate the efficacy and safety of bemarituzumab plus mFOLFOX6 versus placebo plus mFOLFOX6, whereas FORTITUDE-102 will compare bemarituzumab in combination with nivolumab plus physician’s-choice therapy versus nivolumab plus physician’s-choice therapy alone. In addition to the EORTC QLQ-C30 and EQ-5D-5L questionnaires, the studies will also include the 22-question EORTC QLQ-STO22 PRO module specific to gastric cancer, to further evaluate the impact of bemarituzumab on HRQoL.

Strengths and limitations of this analysis must be noted. This is the first study to have explored HRQoL in patients with G/GEJC presenting with FGFR2b-positive cancer using validated, widely used PRO questionnaires. It is also the first study to assess the impact on HRQoL of adding bemarituzumab to chemotherapy as a first-line treatment in advanced G/GEJC. FIGHT was a double-blind trial, which limits the impact of any potential bias on patient responses to the questionnaires. With enrollment of subjects worldwide (57.4% of subjects were recruited in Asian countries), the PRO results capture the perspectives and experiences of patients from diverse geographies. Finally, compliance rates were high and similar between treatment arms both at baseline and during treatment. Nonetheless, FIGHT did not include PRO endpoints in its formal testing scheme; therefore, the analyses presented here were carried out as exploratory *post hoc* analyses. Also, while the EORTC QLQ-C30 does capture the important aspects of HRQoL for patients with G/GEJC, some focused disease-specific aspects captured by stomach cancer questionnaires (e.g. abdominal pain, as measured by the EORTC QLQ-STO22 module) and overall bother from toxicities (e.g. captured by the GP5 question of the Functional Assessment of Cancer Therapy—General [FACT-G] questionnaire) were not reflected. Another limitation is the limited sample size of the FIGHT study; thus, the analyses are associated with an inherent uncertainty, especially in subgroup analyses. To draw more robust conclusions on the impact of adding bemarituzumab to standard-of-care therapy on HRQoL, PRO data from the phase III FORTITUDE-101 and FORTITUDE-102 trials will be leveraged.

Overall, the analysis of the PROs suggested that in patients with previously untreated locally advanced unresectable or metastatic G/GEJC and FGFR2b overexpression treated with bemarituzumab plus mFOLFOX6 and placebo plus mFOLFOX6, HRQoL was generally maintained from baseline, with no meaningful differences between treatment arms. These results complement the meaningful clinical improvements observed with bemarituzumab plus mFOLFOX6 versus placebo plus mFOLFOX6 treatment in the FIGHT study.
