# Supplementary material for: Health-related quality of life with bemarituzumab plus mFOLFOX6 in patients with FGFR2b-overexpressing, advanced gastric or gastroesophageal junction cancer
Source: ESMO Gastrointest Oncol. 2024 Oct 8;6:100095. doi: 10.1016/j.esmogo.2024.100095 (PMC12836807; doi:10.1016/j.esmogo.2024.100095)
Supplement: Supplementary data [file mmc1.docx]

# Supplementary Appendix

Description of PRO measures

### 1. EORTC QLQ-C30

The EORTC QLQ-C30 is a cancer-specific 30-item self-reported questionnaire commonly used in G/GEJC and other cancers that evaluates health status, functioning, and symptoms among individuals. The measure includes a Global Health Status/QoL scale, 5 functional scales (Physical Functioning, Role Functioning, Emotional Functioning, Cognitive Functioning, Social Functioning), 3 symptom scales (Fatigue, Nausea and Vomiting, Pain) and 6 single items (Dyspnea, Insomnia, Appetite Loss, Constipation, Diarrhea, Financial Difficulties). The recall period is the past week. Scale scores range from 0 to 100, with higher scores indicating better HRQOL on the global health status/QoL and functional scales and greater symptom burden on the symptom scales.

### 2. EQ-5D VAS

The EQ-5D-5L is a standardized questionnaire for use as a measure of health outcome developed by the EuroQol group. It comprises a 5-dimension health status measure and a visual analogue scale (VAS). The 5-dimension health status measure is often used to calculate utility indices for economic evaluations and requires the application of societal tariffs to be analyzed. The EQ-5D VAS records a patient’s self-rated current health on a vertical, visual analogue scale, where the endpoints range from 100, “the best health you can imagine,” to 0, “the worst health you can imagine”. For the current analyses evaluating patients’ experiences with treatment, overall health status as measured by the VAS was utilized.

Patient-Reported Outcome Analytical Windows

To account for delayed and/or missed treatment administration and varying end-of-treatment time points, PRO visit dates were classified into the following windows labeled by the week of the target assessment date: week 6, which spanned between day 2 and the target PRO assessment date (day 43) plus 4 weeks (day 71); week 14, which encompassed the period between the target PRO assessment date -4 weeks (day 72) and +4 weeks (day 127); subsequent PRO visits (i.e., week 22, week 30, etc.), which covered the period of ±4 weeks of the target PRO assessment date; and end of treatment, which encompassed the period between the day after the last treatment administration and the following 30 days.

Table S1. Patient-Reported Outcome Analytical Windows

| **PRO Visit per Schedule** | **Target Day** | **Analytical Window** | **Derived PRO visit** |
| --- | --- | --- | --- |
| Screening | - | PRO assessment before first treatment administration | Baseline |
| C4D1 | 43 | Target day – 6 weeks / + 4 weeks [2, 71] | Week 6 |
| C8D1 | 99 | Target day +/- 4 weeks [72, 127] | Week 14 |
| C12D1 | 155 | Target day +/- 4 weeks [128, 183] | Week 22 |
| CXD1 | (2*X-2)*7+1 | Target day +/- 4 weeks [target day-27, min(target day+28, last treatment administration date)] | Week (target day – 1) / 7 |
|  |  |  |  |
| End of treatment | Last treatment administration + 28 days | [last treatment administration + 1, last treatment administration + 31] | End of treatment |

C = cycle; D = day, PRO = patient-reported outcome.

If there were multiple datapoints in the same PRO window, the one closest to the target day was included. If there were two or more assessments equidistant from the target day, the latest one was included.

Table S2. Compliance for the EQ-5D-5L Questionnaire

| **Visit** | **Total (N = 155)** | **Bemarituzumab Arm (N = 77)** | **Placebo Arm (N = 78)** |
| --- | --- | --- | --- |
| Baseline | 149 (96%) | 76 (99%) | 73 (94%) |
| Week 6 | 127/152 (84%) | 65/75 (87%) | 62/77 (81%) |
| Week 14 | 105/119 (88%) | 54/61 (89%) | 51/58 (88%) |
| Week 22 | 73/101 (72%) | 35/49 (71%) | 38/52 (73%) |
| Week 30 | 58/74 (78%) | 34/37 (92%) | 24/37 (65%) |
| Week 38 | 36/49 (73%) | 19/26 (73%) | 17/23 (74%) |
| Week 46 | 31/38 (82%) | 18/22 (82%) | 13/16 (81%) |
| Week 54 | 28/31 (90%) | 17/19 (89%) | 11/12 (92%) |
| Week 62 | 17/23 (74%) | 10/13 (77%) | 7/10 (70%) |
| Week 70 | 13/18 (72%) | 8/10 (80%) | 5/8 (63%) |
| Week 78 | 10/15 (67%) | 4/8 (50%) | 6/7 (86%) |
| Week 86 | 11/13 (85%) | 5/6 (83%) | 6/7 (86%) |
| Week 94 | 8/12 (67%) | 6/6 (100%) | 2/6 (33%) |
| Week 102 | 7/8 (88%) | 4/4 (100%) | 3/4 (75%) |
| Week 110 | 5/5 (100%) | 4/4 (100%) | 1/1 (100%) |
| Week 118 | 4/5 (80%) | 3/4 (75%) | 1/1 (100%) |
| Week 126 | 4/5 (80%) | 4/4 (100%) | 0/1 (0%) |
| Week 134 | 3/3 (100%) | 3/3 (100%) | 0 |
| Week 142 | 1/2 (50%) | 1/2 (50%) | 0 |
| Week 150 | 1/1 (100%) | 1/1 (100%) | 0 |
| End of treatment | 62/152 (41%) | 28/75 (37%) | 34/77 (44%) |

Note: Less than 15% of the ITT population was treated hence expected to have a PRO assessment after week 54.

Table S3. Mixed Model for Repeated Measures Change From Baseline in PRO Scores

| **Scale** | **Week** | **LS Mean Change From Baseline (95% CI)** | | **Difference (95% CI)** |
| --- | --- | --- | --- | --- |
|  |  | **Bemarituzumab (N = 77)** | **Placebo  (N = 78)** |  |
| *EORTC QLQ-C30* |  |  |  |  |
| Global Health Status/QOL | Week 6 | 6.4 (2.4, 10.4) | 6.2 (2.0, 10.5) | 0.2 (-5.6, 6.0) |
|  | Week 14 | 4.8 (0.9, 8.8) | 3.7 (-0.6, 7.9) | 1.1 (-4.6, 6.9) |
|  | Week 22 | 7.9 (2.5, 13.4) | 2.5 (-2.9, 7.9) | 5.4 (-2.3, 13.1) |
|  | Week 30 | 2.2 (-5.1, 9.4) | 3.0 (-5.7, 11.6) | -0.8 (-12.1, 10.4) |
|  | Week 38 | 1.8 (-5.9, 9.6) | -5.1 (-13.8, 3.7) | 6.9 (-4.8, 18.6) |
|  | Week 46 | -1.2 (-9.2, 6.8) | 0.7 (-9.4, 10.8) | -2.0 (-14.8, 10.9) |
|  | Week 54 | -1.1 (-12.7, 10.4) | -1.7 (-17.0, 13.7) | 0.5 (-18.6, 19.7) |
| Physical Functioning | Week 6 | 1.5 (-1.2, 4.3) | -0.3 (-3.2, 2.6) | 1.8 (-2.2, 5.9) |
|  | Week 14 | -0.2 (-3.7, 3.2) | -1.3 (-5.0, 2.4) | 1.1 (-4.0, 6.1) |
|  | Week 22 | -0.8 (-5.7, 4.0) | -7.1 (-12.0, -2.3) | 6.3 (-0.6, 13.2) |
|  | Week 30 | -7.1 (-12.7, -1.5) | -8.2 (-14.7, -1.8) | 1.1 (-7.4, 9.6) |
|  | Week 38 | -10.9 (-16.9, -4.8) | -9.1 (-15.7, -2.5) | -1.8 (-10.7, 7.2) |
|  | Week 46 | -8.7 (-14.9, -2.5) | -7.9 (-15.2, -0.6) | -0.8 (-10.4, 8.8) |
|  | Week 54 | -10.9 (-18.5, -3.2) | -9.6 (-19.0, -0.3) | -1.2 (-13.3, 10.8) |
| Role Functioning | Week 6 | 2.3 (-2.0, 6.7) | -5.8 (-10.4, -1.2) | 8.1 (1.8, 14.4) |
|  | Week 14 | 3.8 (-1.1, 8.7) | -6.2 (-11.4, -1.0) | 10.0 (2.9, 17.2) |
|  | Week 22 | -3.6 (-10.8, 3.6) | -10.5 (-17.6, -3.3) | 6.8 (-3.3, 17.0) |
|  | Week 30 | -6.6 (-15.5, 2.4) | -12.6 (-23.0, -2.2) | 6.0 (-7.6, 19.7) |
|  | Week 38 | -9.2 (-18.4, -0.1) | -12.9 (-23.0, -2.9) | 3.7 (-9.9, 17.3) |
|  | Week 46 | -6.5 (-14.0, 1.1) | -7.2 (-16.2, 1.8) | 0.7 (-11.0, 12.5) |
|  | Week 54 | -10.6 (-22.0, 0.7) | -8.0 (-22.1, 6.2) | -2.7 (-20.8, 15.4) |
| Emotional Functioning | Week 6 | 9.2 (6.2, 12.1) | 2.8 (-0.4, 6.0) | 6.4 (2.0, 10.7) |
|  | Week 14 | 6.2 (2.2, 10.1) | 2.1 (-2.1, 6.4) | 4.0 (-1.8, 9.8) |
|  | Week 22 | 8.2 (3.9, 12.5) | -0.9 (-5.3, 3.4) | 9.1 (3.0, 15.3) |
|  | Week 30 | 6.2 (1.2, 11.1) | -3.9 (-9.7, 2.0) | 10.0 (2.4, 17.6) |
|  | Week 38 | 3.4 (-3.3, 10.0) | -7.3 (-14.8, 0.2) | 10.6 (0.7, 20.6) |
|  | Week 46 | 3.6 (-2.3, 9.6) | -0.4 (-7.9, 7.2) | 4.0 (-5.6, 13.6) |
|  | Week 54 | 1.5 (-6.7, 9.7) | -9.6 (-20.3, 1.0) | 11.1 (-2.2, 24.4) |
| Cognitive Functioning | Week 6 | 1.2 (-1.7, 4.2) | -2.1 (-5.3, 1.1) | 3.3 (-1.0, 7.7) |
|  | Week 14 | -2.5 (-6.3, 1.2) | -3.9 (-7.9, 0.1) | 1.4 (-4.1, 6.8) |
|  | Week 22 | -0.6 (-5.0, 3.7) | -3.5 (-7.9, 1.0) | 2.8 (-3.4, 9.0) |
|  | Week 30 | -7.9 (-12.5, -3.2) | -3.0 (-8.5, 2.5) | -4.8 (-12.0, 2.3) |
|  | Week 38 | -8.7 (-15.5, -1.8) | -7.3 (-15.0, 0.3) | -1.3 (-11.6, 8.9) |
|  | Week 46 | -12.6 (-19.9, -5.4) | -9.6 (-18.7, -0.4) | -3.1 (-14.7, 8.6) |
|  | Week 54 | -15.9 (-24.8, -6.9) | -8.6 (-20.4, 3.2) | -7.3 (-22.0, 7.3) |
| Social Functioning | Week 6 | 8.2 (3.7, 12.7) | -2.2 (-7.0, 2.5) | 10.4 (3.9, 17.0) |
|  | Week 14 | 4.3 (-0.4, 8.9) | -3.4 (-8.3, 1.6) | 7.6 (0.9, 14.4) |
|  | Week 22 | -0.3 (-6.9, 6.4) | -7.2 (-13.9, -0.5) | 6.9 (-2.5, 16.4) |
|  | Week 30 | 2.5 (-4.5, 9.6) | -5.9 (-14.3, 2.4) | 8.5 (-2.4, 19.4) |
|  | Week 38 | -16.4 (-26.9, -5.9) | -7.2 (-19.0, 4.6) | -9.2 (-25.0, 6.6) |
|  | Week 46 | -5.8 (-15.3, 3.6) | -6.8 (-18.7, 5.1) | 1.0 (-14.2, 16.1) |
|  | Week 54 | -7.0 (-19.7, 5.8) | -5.2 (-21.9, 11.5) | -1.8 (-22.7, 19.2) |
| Fatigue | Week 6 | -0.4 (-4.4, 3.7) | -2.4 (-6.7, 1.9) | 2.0 (-3.9, 7.9) |
|  | Week 14 | -0.5 (-5.5, 4.5) | 3.6 (-1.7, 8.9) | -4.1 (-11.4, 3.1) |
|  | Week 22 | 1.1 (-3.8, 6.0) | 4.3 (-0.6, 9.1) | -3.2 (-10.1, 3.7) |
|  | Week 30 | 1.6 (-3.9, 7.0) | 4.2 (-2.2, 10.6) | -2.7 (-11.0, 5.7) |
|  | Week 38 | 6.9 (0.1, 13.6) | 4.9 (-2.4, 12.2) | 1.9 (-8.0, 11.9) |
|  | Week 46 | 6.5 (-1.5, 14.6) | 3.1 (-6.7, 12.8) | 3.4 (-9.2, 16.1) |
|  | Week 54 | 4.6 (-4.3, 13.4) | 5.5 (-5.6, 16.6) | -0.9 (-15.0, 13.1) |
| Nausea and Vomiting | Week 6 | -1.7 (-5.3, 1.9) | -2.5 (-6.3, 1.3) | 0.8 (-4.4, 6.1) |
|  | Week 14 | -0.8 (-5.5, 3.9) | 1.4 (-3.6, 6.4) | -2.2 (-9.0, 4.7) |
|  | Week 22 | -1.5 (-7.8, 4.8) | 3.1 (-3.3, 9.4) | -4.6 (-13.5, 4.3) |
|  | Week 30 | -4.1 (-9.1, 0.8) | -1.2 (-7.0, 4.5) | -2.9 (-10.4, 4.7) |
|  | Week 38 | 0.1 (-6.1, 6.3) | -0.7 (-7.4, 6.1) | 0.8 (-8.4, 10.0) |
|  | Week 46 | -2.6 (-9.5, 4.3) | -1.2 (-9.5, 7.1) | -1.4 (-12.2, 9.4) |
|  | Week 54 | 2.1 (-3.7, 8.0) | -2.7 (-10.0, 4.6) | 4.8 (-4.5, 14.1) |
| Pain | Week 6 | -7.1 (-11.0, -3.3) | -6.0 (-10.0, -1.9) | -1.2 (-6.8, 4.4) |
|  | Week 14 | -5.0 (-9.7, -0.2) | -0.8 (-5.8, 4.2) | -4.2 (-11.1, 2.7) |
|  | Week 22 | 0.8 (-6.2, 7.9) | 2.8 (-4.1, 9.8) | -2.0 (-11.9, 7.9) |
|  | Week 30 | 0.7 (-5.5, 6.8) | -4.2 (-11.6, 3.1) | 4.9 (-4.6, 14.4) |
|  | Week 38 | 8.0 (-0.3, 16.3) | -3.2 (-12.2, 5.8) | 11.2 (-1.0, 23.5) |
|  | Week 46 | 8.6 (-0.1, 17.3) | 0.7 (-9.7, 11.2) | 7.9 (-5.7, 21.5) |
|  | Week 54 | 6.9 (-3.3, 17.0) | 0.3 (-12.4, 13.0) | 6.6 (-9.6, 22.8) |
| Dyspnoea | Week 6 | 0.4 (-3.3, 4.2) | 0.6 (-3.4, 4.6) | -0.2 (-5.7, 5.3) |
|  | Week 14 | 1.8 (-2.4, 6.0) | -0.4 (-4.8, 4.1) | 2.2 (-3.9, 8.3) |
|  | Week 22 | -1.4 (-6.8, 3.9) | 3.9 (-1.4, 9.2) | -5.3 (-12.9, 2.2) |
|  | Week 30 | 1.5 (-3.6, 6.5) | 3.4 (-2.7, 9.4) | -1.9 (-9.7, 6.0) |
|  | Week 38 | 4.8 (-1.6, 11.2) | 3.9 (-3.0, 10.8) | 0.9 (-8.5, 10.3) |
|  | Week 46 | 5.7 (-3.3, 14.7) | 8.0 (-2.9, 18.9) | -2.3 (-16.4, 11.8) |
|  | Week 54 | 4.5 (-1.3, 10.3) | -0.9 (-8.2, 6.4) | 5.4 (-3.9, 14.6) |
| Insomnia | Week 6 | -6.5 (-11.1, -1.8) | -7.2 (-12.1, -2.3) | 0.7 (-6.0, 7.5) |
|  | Week 14 | -9.9 (-16.0, -3.8) | -3.9 (-10.4, 2.5) | -6.0 (-14.8, 2.9) |
|  | Week 22 | -0.2 (-8.2, 7.9) | 1.8 (-6.2, 9.7) | -1.9 (-13.2, 9.4) |
|  | Week 30 | -5.7 (-12.2, 0.9) | -8.4 (-16.2, -0.5) | 2.7 (-7.5, 12.9) |
|  | Week 38 | -4.8 (-13.1, 3.5) | -10.2 (-19.2, -1.2) | 5.4 (-6.8, 17.6) |
|  | Week 46 | -0.2 (-10.7, 10.2) | -8.9 (-21.6, 3.8) | 8.7 (-7.7, 25.1) |
|  | Week 54 | -1.3 (-13.7, 11.2) | 2.3 (-13.5, 18.1) | -3.6 (-23.6, 16.5) |
| Appetite Loss | Week 6 | -4.8 (-10.4, 0.8) | -6.8 (-12.7, -0.9) | 2.0 (-6.2, 10.1) |
|  | Week 14 | -7.3 (-14.0, -0.5) | 0.9 (-6.3, 8.0) | -8.1 (-18.0, 1.7) |
|  | Week 22 | -5.8 (-14.2, 2.7) | 1.7 (-6.6, 10.1) | -7.5 (-19.3, 4.3) |
|  | Week 30 | -8.2 (-15.7, -0.6) | -10.6 (-19.6, -1.7) | 2.5 (-9.2, 14.2) |
|  | Week 38 | -11.8 (-21.0, -2.7) | -5.6 (-15.5, 4.4) | -6.3 (-19.8, 7.3) |
|  | Week 46 | -11.8 (-21.3, -2.4) | -0.7 (-12.1, 10.8) | -11.2 (-26.0, 3.6) |
|  | Week 54 | -8.1 (-17.0, 0.8) | -7.8 (-19.0, 3.4) | -0.3 (-14.6, 14.0) |
| Constipation | Week 6 | -5.4 (-10.9, 0.2) | 7.0 (1.1, 12.8) | -10.0 (-18.9, -1.2) |
|  | Week 14 | -3.8 (-9.9, 2.3) | 6.3 (-0.2, 12.7) | -10.0 (-18.9, -1.2) |
|  | Week 22 | -3.6 (-11.1, 4.0) | 8.5 (1.2, 15.9) | -12.1 (-22.6, -1.6) |
|  | Week 30 | -2.4 (-10.3, 5.4) | 4.2 (-5.0, 13.4) | -6.6 (-18.7, 5.5) |
|  | Week 38 | 2.3 (-8.0, 12.6) | 2.5 (-9.6, 14.6) | -0.2 (-16.0, 15.7) |
|  | Week 46 | 4.2 (-9.4, 17.8) | 1.5 (-15.7, 18.7) | 2.7 (-19.1, 24.5) |
|  | Week 54 | 5.0 (-4.1, 14.2) | 4.7 (-3.7, 13.1) | 0.4 (-12.1, 12.8) |
| Diarrhea | Week 6 | 0.9 (-3.7, 5.6) | 2.3 (-2.7, 7.3) | -1.4 (-8.2, 5.5) |
|  | Week 14 | -0.6 (-4.9, 3.7) | -0.5 (-5.1, 4.1) | -0.0 (-6.3, 6.2) |
|  | Week 22 | -2.9 (-8.5, 2.8) | -3.5 (-9.1, 2.1) | 0.7 (-7.3, 8.6) |
|  | Week 30 | -1.5 (-7.7, 4.7) | -5.2 (-12.7, 2.2) | 3.7 (-6.0, 13.4) |
|  | Week 38 | -1.3 (-9.5, 7.0) | 2.2 (-7.0, 11.5) | -3.5 (-15.9, 8.9) |
|  | Week 46 | -3.6 (-12.1, 4.8) | 15.9 (5.1, 26.6) | -19.5 (-33.2, -5.8) |
|  | Week 54 | -1.5 (-9.0, 6.0) | 1.0 (-9.0, 11.0) | -2.5 (-14.9, 10.0) |
| Financial Difficulties | Week 6 | -1.9 (-7.3, 3.4) | -1.7 (-7.4, 4.0) | -0.2 (-8.0, 7.6) |
|  | Week 14 | 1.3 (-4.4, 7.0) | -0.5 (-6.5, 5.6) | 1.8 (-6.6, 10.1) |
|  | Week 22 | 0.9 (-6.5, 8.3) | 6.9 (-0.6, 14.4) | -6.0 (-16.5, 4.5) |
|  | Week 30 | 5.4 (-3.2, 14.0) | 3.9 (-6.2, 14.1) | 1.5 (-11.7, 14.7) |
|  | Week 38 | 6.1 (-4.3, 16.4) | 5.2 (-6.4, 16.8) | 0.9 (-14.6, 16.4) |
|  | Week 46 | 4.5 (-8.0, 17.1) | 9.1 (-6.8, 24.9) | -4.5 (-24.7, 15.6) |
|  | Week 54 | 13.3 (0.2, 26.4) | 2.0 (-15.2, 19.2) | 11.3 (-10.1, 32.7) |
| *EQ-5D VAS* |  |  |  |  |
| Scale | Week 6 | 6.8 (3.8, 9.9) | 2.4 (-0.8, 5.7) | 4.4 (-0.1, 8.8) |
|  | Week 14 | 8.5 (5.3, 11.7) | 2.3 (-1.2, 5.7) | 6.3 (1.5, 11.0) |
|  | Week 22 | 4.8 (-0.6, 10.1) | 2.6 (-2.7, 7.9) | 2.2 (-5.3, 9.7) |
|  | Week 30 | 0.4 (-4.8, 5.7) | 0.4 (-5.9, 6.6) | 0.1 (-8.1, 8.3) |
|  | Week 38 | 2.2 (-3.9, 8.3) | 3.2 (-3.4, 9.8) | -1.0 (-10.0, 8.0) |
|  | Week 46 | -1.0 (-7.9, 5.9) | 1.7 (-6.5, 9.8) | -2.7 (-13.3, 8.0) |
|  | Week 54 | 1.0 (-6.2, 8.1) | 7.6 (-1.3, 16.4) | -6.6 (-18.0, 4.7) |

CI = confidence interval; LS = least squares; PRO = patient-reported outcome; VAS = visual analogue scale.

Results are based on a MMRM model on PRO change from baseline with treatment, baseline score, analysis visit, treatment-by-analysis visit interaction, and randomization stratification factors as covariates. Heterogeneous Toeplitz covariance structure was used in the model to account for the within-patient correlations. Analyses were limited to study assessments with at least 10 patients with evaluable data in each treatment arm.

EORTC QLQ-C30 scale scores range from 0 to 100, with higher scores indicating better HRQOL on the global and functional scales and greater symptom burden on the symptom scales. EQ-5D VAS score ranges from 100, “the best health you can imagine,” to 0, “the worst health you can imagine.”

Figure S1. Least-Squares Mean Changes From Baseline in PRO Scores on the EORTC QLQ-C30 Global Health Status/QoL Scale, by Subgroup

**a. Patients with 2+/3+ FGFR2b IHC staining intensity in ≥10% of tumor cells**


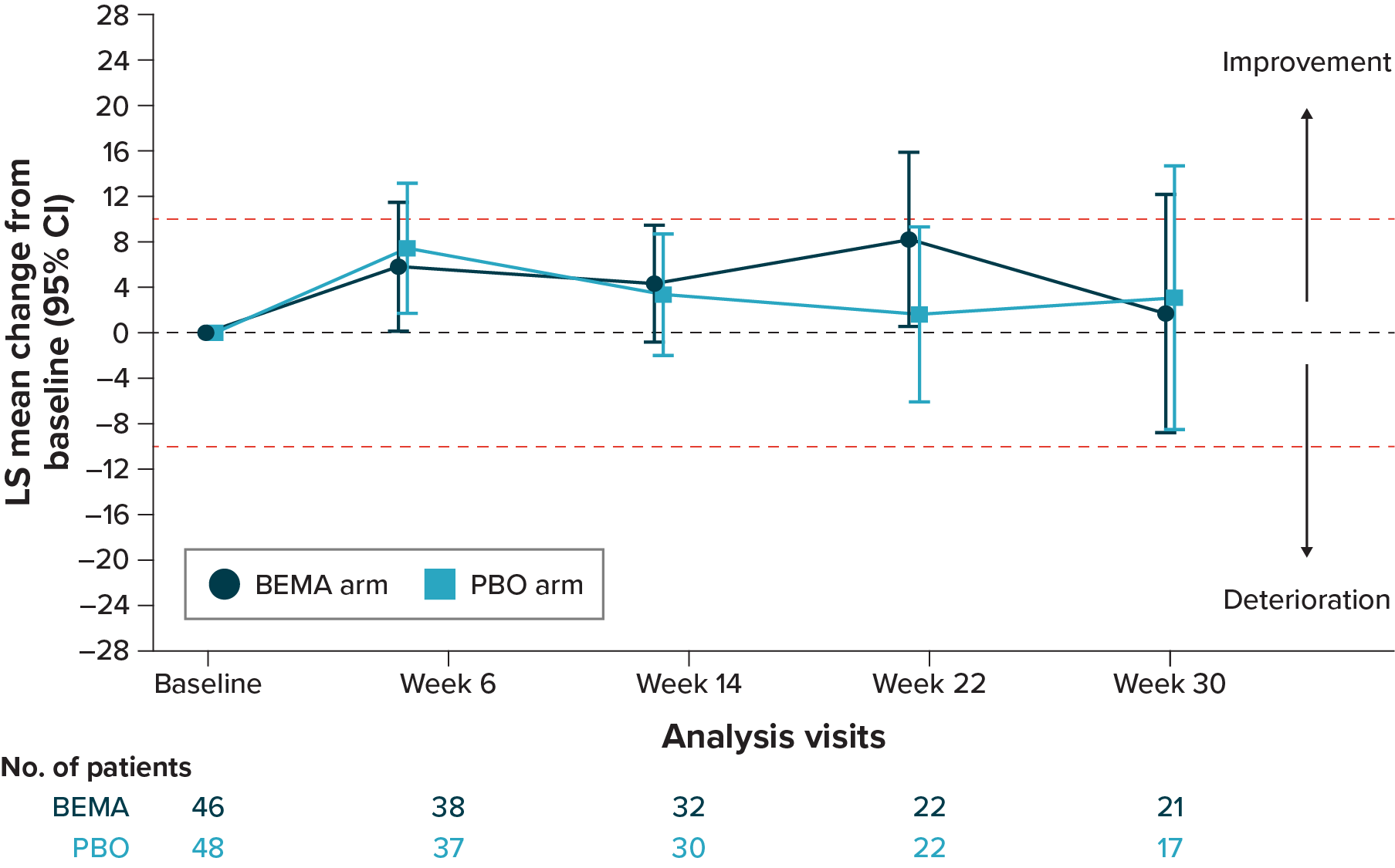


**b. Patients with Asian geographical location**


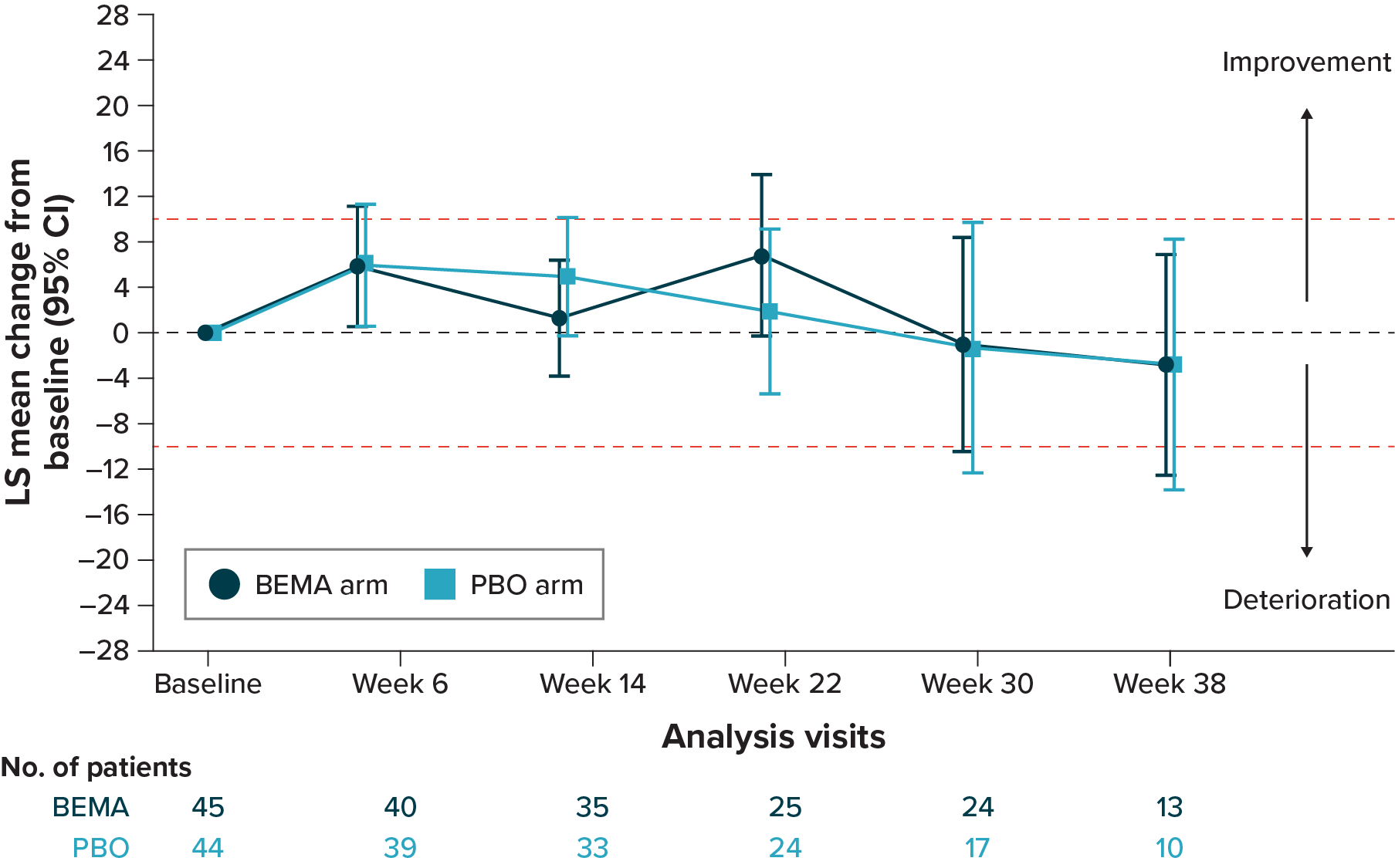


**c. Patients with non-Asian geographical location**


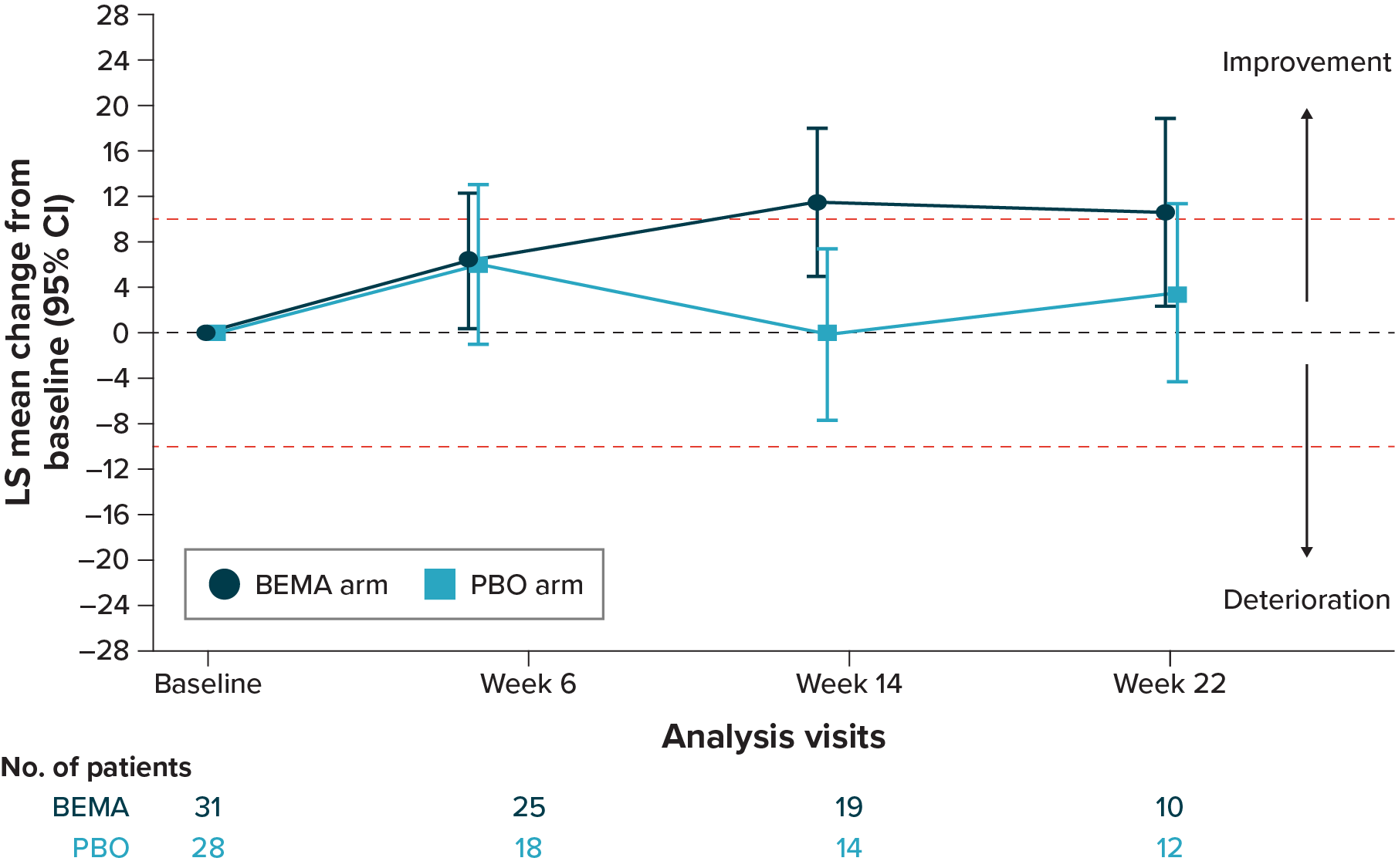


EORTC QLQ-C30: European Organisation for the Research and Treatment of Cancer Quality of Life Questionnaire; HRQOL = health-related quality of life.

Red dotted lines indicate thresholds for meaningful change from baseline. EORTC QLQ-C30 scale scores range from 0 to 100, with higher scores indicating better HRQOL on the global and functional scales and greater symptom burden on the symptom scales. EQ-5D VAS score ranges from 100, “the best health you can imagine,” to 0, “the worst health you can imagine.”

Figure S2. Time to Sustained Deterioration


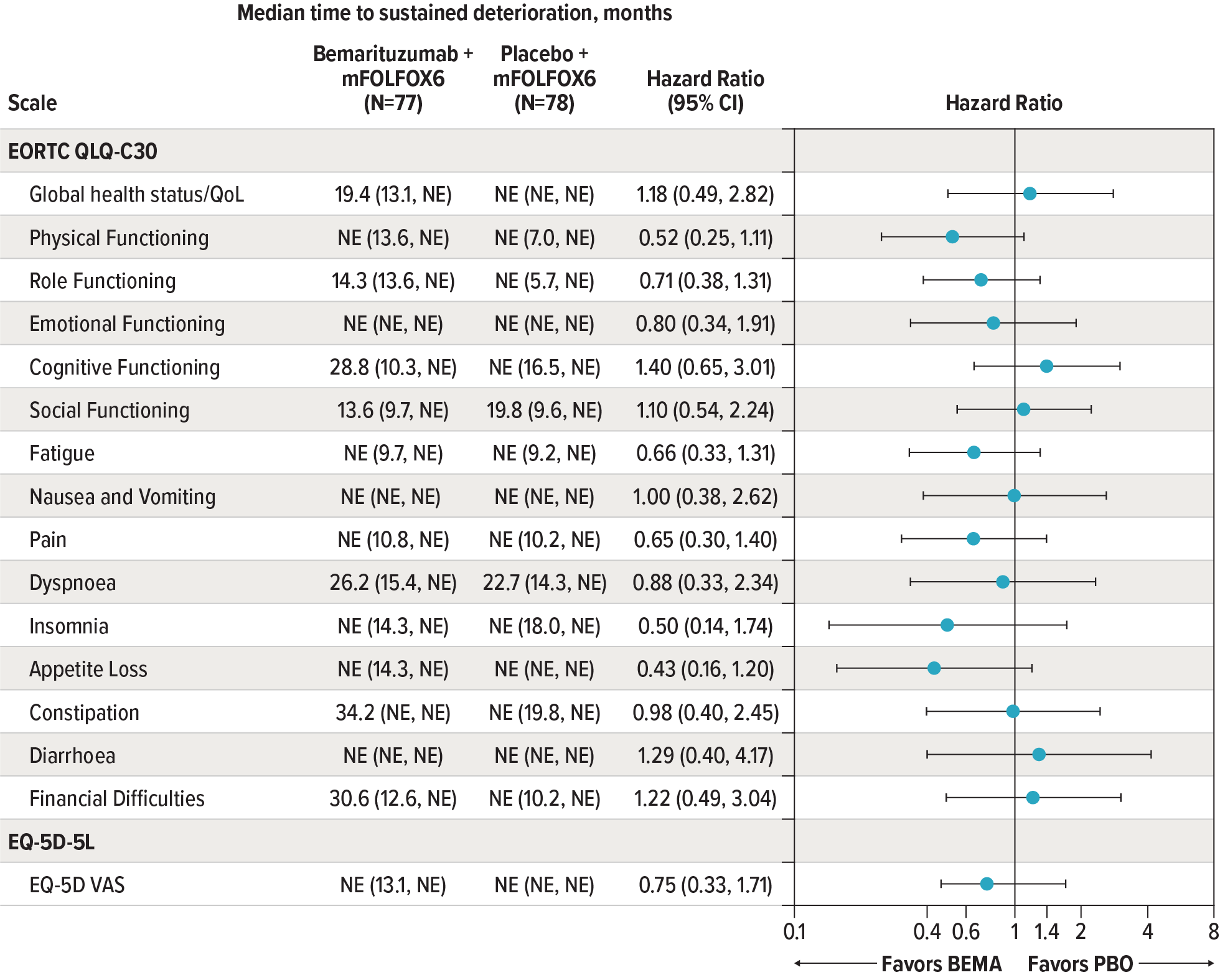


BEMA = bemarituzumab; NE = not estimable; PBO = placebo.

Hazard ratios are presented in log scale plots.
